# MAP3K4 signaling regulates HDAC6 and TRAF4 coexpression and stabilization in trophoblast stem cells

**DOI:** 10.1016/j.jbc.2024.108116

**Published:** 2024-12-20

**Authors:** Hannah A. Nelson, Nathan A. Mullins, Amy N. Abell

**Affiliations:** Department of Biological Sciences, University of Memphis, Memphis, Tennessee, USA

**Keywords:** TRAF4, HDAC6, MAP3K4, trophoblast stem cell, placenta, protein complex, protein expression, protein-protein interaction, protein stability

## Abstract

Mitogen-activated protein kinase kinase kinase 4 (MAP3K4) promotes fetal and placental growth and development, with MAP3K4 kinase inactivation resulting in placental insufficiency and fetal growth restriction. MAP3K4 promotes key signaling pathways including JNK, p38, and PI3K/Akt, leading to activation of CREB-binding protein. MAP3K4 kinase inactivation results in loss of these pathways and gain of histone deacetylase 6 (HDAC6) expression and activity. Tumor necrosis factor receptor-associated factor 4 (TRAF4) binds MAP3K4 and promotes MAP3K4 activation of downstream pathways in the embryo; however, the role of TRAF4 and its association with MAP3K4 in the placenta is unknown. Our analyses of murine placenta single-cell RNA-Seq data showed that *Traf4* is coexpressed with *Map3k4* in trophoblast stem (TS) cells and labyrinth progenitors, whereas *Hdac6* expression is higher in differentiated trophoblasts. We demonstrate that, like HDAC6, TRAF4 expression is increased in MAP3K4 kinase-inactive TS (TS^KI^) cells and upon inhibition of MAP3K4-dependent pathways in WT TS cells. Moreover, *Hdac6* shRNA knockdown in TS^KI^ cells reduces TRAF4 protein expression. We found that HDAC6 forms a protein complex with TRAF4 in TS cells and promotes TRAF4 expression in the absence of HDAC6 deacetylase activity. Finally, we examine the relationships among MAP3K4, TRAF4, and HDAC6 in the developing placenta, finding a previously unknown switch in the coexpression of *Traf4* with *Map3k4 versus Traf4* with *Hdac6* during differentiation of the placental labyrinth. Together, our findings identify previously unknown mechanisms of MAP3K4 and HDAC6 coregulation of TRAF4 in TS cells and highlight these MAP3K4, TRAF4, and HDAC6 associations during placental development.

The placenta is an extraembryonic organ critical for fetal growth and development that transports nutrients, oxygen, and hormones to the developing fetus. Placental insufficiency or dysfunction can negatively impact fetal growth (1, 2). The placenta is formed from trophoblast stem (TS) cells derived from the trophectoderm of the blastocyst, endothelial cells derived from the fetus, and decidual cells derived from the mother (3). TS cells give rise to all trophoblast subtypes in the placenta, which constitute the majority of placental cells. TS cells can be cultured indefinitely in the presence of stemness-maintaining factors, whereas removal induces differentiation of TS cells to all trophoblast subtypes (4, 5).

Mitogen-activated protein kinase kinase kinase 4 (MAP3K4) is a member of the MAP3K family of protein kinases. MAP3K4 is abundantly expressed in TS cells (5). Signaling through MAP3K4 regulates processes including epithelial-to-mesenchymal transition and embryonic and placental growth (6, 7, 8, 9, 10). Upon activation, MAP3K4 activates the mitogen-activated protein kinase kinases (MAP2Ks) MAP2K3/6 and MAP2K4/7, resulting in activation of the mitogen-activated protein kinases (MAPKs) p38 and c-Jun N-terminal kinase (JNK) (5, 7). A point mutation in the active-site lysine at amino acid 1361 to arginine renders MAP3K4 kinase-inactive (KI), resulting in the loss of MAP3K4 enzymatic activity and downstream signaling (7). MAP3K4 KI TS cells (TS^KI^ cells) exhibit properties of epithelial-to-mesenchymal transition, including the acquisition of mesenchymal characteristics and invasiveness, due in part to increased expression and activity of histone deacetylase 6 (HDAC6) (5, 6, 8). Mice homozygous for MAP3K4 kinase inactivation (*Map3k4*^*KI/KI*^) exhibit highly penetrant lethality prior to weaning and embryonic defects including exencephaly, spina bifida, and fetal growth restriction (7, 9). Loss of MAP3K4 kinase activity also results in placental defects including trophoblast hyperinvasion leading to defects in implantation and reduced placental size and weight (6, 7, 9). These defects demonstrate the importance of MAP3K4-dependent pathways on normal fetal and placental growth and development.

Tumor necrosis factor receptor-associated factor 4 (TRAF4) is a MAP3K4 interacting partner and atypical member of the TRAF family of adaptor proteins (11, 12). As adaptors, the TRAFs transduce signals from upstream receptors to downstream signaling molecules (13). Unlike other TRAF family members, TRAF4 does not interact with canonical members of the tumor necrosis factor receptor family, and the receptor-recognition motifs in the TRAF domain of TRAF4 differ significantly from other TRAF members (14, 15, 16). *TRAF4* was initially identified as an amplified gene in human breast carcinoma, and overexpression of TRAF4 protein occurs in other human cancers (17, 18, 19). TRAF4 promotes the proliferation, invasion, and survival of cancer through the Akt, Wnt/β-catenin, and MAPK pathways (20, 21, 22, 23, 24). TRAF4 is also critical for embryonic development. In mice, *Traf4* is expressed at high levels during embryogenesis (25). Knockout of *Traf4* results in incompletely penetrant embryonic lethality and developmental defects including spina bifida, skeletal malformations, tracheal abnormalities, and reduced growth (26, 27). Although TRAF4 is important during development, studies investigating the specific signaling partners and molecular mechanisms of TRAF4 regulation of these developmental processes are lacking.

Our lab has previously shown that TRAF4 promotes MAP3K4 dimerization and activity (11). The TRAF domain of TRAF4 interacts with the kinase domain of MAP3K4, enhancing MAP3K4 dimerization and downstream JNK activation (11). Significantly, MAP3K4 and TRAF4 are highly expressed and interact endogenously in the developing mouse embryo, and *Traf4*^*−/−*^ mice and *Map3k4*^*KI/KI*^ mice exhibit overlapping developmental phenotypes (7, 9, 11, 26, 27). The endogenous interaction and overlapping phenotypes of *Traf4*^*−/−*^ and *Map3k4*^*KI/KI*^ mice suggest that TRAF4 and MAP3K4 function in developmentally important common pathways. However, unlike MAP3K4, a role for TRAF4 in placental development has not been previously shown.

To identify a potential role for TRAF4 in the placenta, we examined *Traf4* in trophoblasts in the placenta. We discovered that *Traf4* is one of the most abundant Trafs in the mouse placenta and is concomitantly expressed with *Map3k4* in TS cells and specific labyrinth progenitors. In contrast, *Hdac6* is relatively weakly expressed in TS cells but is higher in differentiated trophoblasts. Similar to HDAC6, TRAF4 expression is elevated in TS^KI^ cells and upon inhibition of MAP3K4-dependent signaling pathways. shRNA knockdown of HDAC6 in TS^KI^ cells reduces TRAF4 expression. We found that HDAC6 stabilizes TRAF4 and forms a complex with TRAF4 that does not require HDAC6 deacetylase activity, demonstrating a previously unknown role of HDAC6 as a TRAF4 regulator and binding partner. Intriguingly, we show that coexpression of *Traf4* with either *Map3k4* or *Hdac6* changes during placental differentiation, particularly in cell types of the labyrinth. These findings suggest potential normal roles for MAP3K4, TRAF4, and HDAC6 in labyrinth formation during development that may be dysregulated with MAP3K4 kinase inactivation.

## Results

### Coexpression of Map3k4 and Traf4 in TS cells and labyrinth progenitors

**Figure 1:**
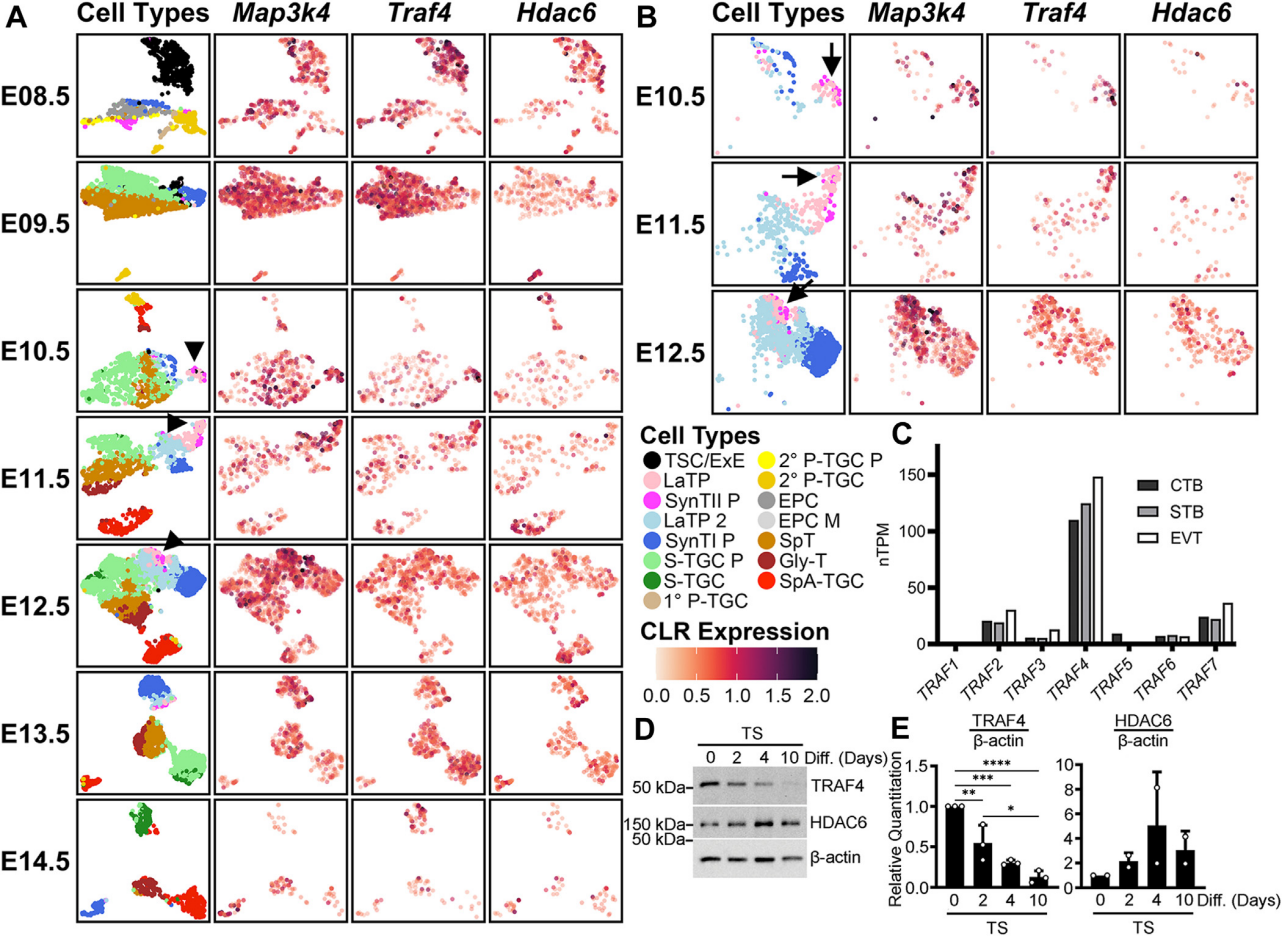
***Traf4* and *Map3k4* are coexpressed in TS cells and labyrinth progenitors.***A* and *B*, uniform manifold approximation and projection (UMAP) plots show single-cell RNA-seq gene expression patterns in mouse placenta during embryonic (E) development. *Arrowheads* in *A* indicate inset locations. *B*, insets show gene expression in LaTP, LaTP 2, SynTI P, and SynTII P cells. *Arrows* in *B* indicate cell types with robust *Traf4* and *Map3k4* coexpression. *C*, *TRAF4* is the most abundant TRAF in human trophoblasts. Data show normalized transcripts per million (nTPM) from the Human Protein Atlas. *D* and *E*, TRAF4 protein expression is highest in undifferentiated TS cells and decreases with differentiation, whereas HDAC6 expression increases with differentiation. Data show (*D*) representative Western blots and (*E*) densitometry analyses normalized to β-actin. *E*, TRAF4 bar graphs are the mean ± SD of three biologically independent experiments. HDAC6 bar graphs in *E* are the mean ± range of two biologically independent experiments. ∗ *p*-value < 0.05; ∗∗ *p*-value < 0.01; ∗∗∗ *p*-value < 0.001; ∗∗∗∗ *p*-value < 0.0001; one-way ANOVA with Tukey’s HSD *post hoc* test. CLR, centered log-ratio; CTB, cytotrophoblasts; EPC, ectoplacental cone; EPC M, EPC migratory; EVT, extravillous trophoblasts; ExE, extraembryonic ectoderm; Gly-T, glycogen trophoblasts; HDAC6, histone deacetylase 6; LaTP, labyrinth trophoblast progenitor; P-TGC, parietal TGC; P-TGC P, P-TGC precursor; STB, human syncytiotrophoblasts; S-TGC, sinusoidal TGC; S-TGC P, S-TGC precursor; SpA-TGC, spiral-artery TGC; SpT, spongiotrophoblast; SynT, syncytiotrophoblast; SynTI P, SynT layer I precursor; SynTII P, SynT layer II precursor; TGC, trophoblast giant cell; TS, trophoblast stem; TSC, TS cell; 1°, primary; 2° secondary.

TRAF4 is a MAP3K4-binding partner in early embryos, promoting MAP3K4 homodimerization and JNK activation (11). In addition to its role in embryonic development, MAP3K4 is highly expressed in TS cells that form placental trophoblasts (5, 6, 8, 9). To identify potential roles for TRAF4 in the placenta, we examined the expression of *Traf4* transcript during murine placental development using published single-cell RNA-Seq (scRNAseq) data (28). *Map3k4* and *Traf4* were among the most abundant Map3ks and Trafs, respectively, in the placenta (Figs. S1 and S2). *Map3k4* and *Traf4* expression was highest during early placental development with coexpression in the TS cells and labyrinth progenitors (Fig. 1, *A* and *B*). *TRAF4* was also the most abundant TRAF in human trophoblasts, suggesting a possible role for TRAF4 in human placentas (Fig. 1*C*). Consistent with *in vivo* scRNAseq data, TRAF4 protein was highly expressed in cultured TS cells, and TRAF4 levels decreased during *in vitro* differentiation (Fig. 1, *D* and *E*). These data suggest a possible role for TRAF4 in TS cells and during early placental development.

We also examined the expression of *Hdac6* during placental development. HDAC6 is negatively regulated by MAP3K4 in TS cells, as WT MAP3K4 binds HDAC6 and promotes HDAC6 ubiquitination and degradation (8). While KI MAP3K4 maintains the ability to bind HDAC6, inactivation of MAP3K4 disrupts HDAC6 ubiquitination and degradation, resulting in overexpression and hyperactivation of HDAC6 in TS^KI^ cells (8). Unlike *Map3k4* and *Traf4*, *Hdac6* was expressed at relatively low levels in normal placental TS cells and labyrinth progenitors and instead appeared more abundant in more differentiated cells of the labyrinth (Fig. 1, *A* and *B*). Similarly, HDAC6 protein expression increased with *in vitro* differentiation of TS cells, consistent with our previously published findings (Fig. 1, *D* and *E*) (8). These data suggest that though HDAC6 is overexpressed in TS cells lacking MAP3K4 activity, *Hdac6* is expressed at relatively low levels in TS cells during normal development and instead is abundant in more differentiated trophoblasts. In contrast, *Traf4* is abundant and coexpressed with *Map3k4* in TS cells and labyrinth progenitors during normal placental development.

### TRAF4 expression in TS cells is coregulated by MAP3K4 and HDAC6

**Figure 2:**
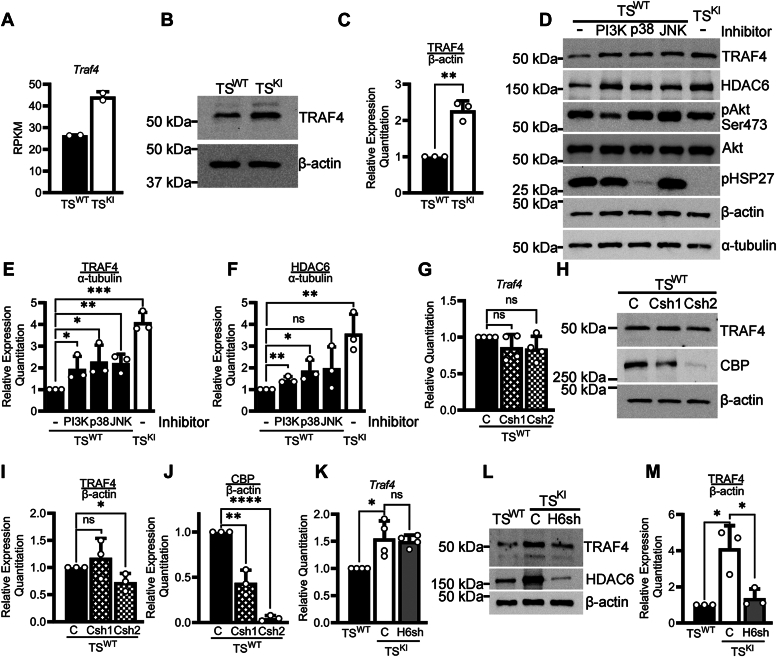
**TRAF4 expression in TS cells is MAP3K4- and HDAC6-dependent.***A–C*, *Traf4* expression is elevated in MAP3K4 kinase-inactive TS cells (TS^KI^ cells) relative to WT TS (TS^WT^) cells. *A,* bar graphs show *Traf4* transcript measured by RNA-Seq and are the mean ± range of two biologically independent experiments. *B*, representative Western blots and (*C*) densitometry analyses of blots are shown. *D–F*, inhibition of MAP3K4-dependent pathways in TS^WT^ cells increases TRAF4 protein expression. Cells were treated with either vehicle control (DMSO) or inhibitors for the pathways shown for 24 h. *D*, representative Western blots and (*E* and *F*) densitometry analyses normalized to α-tubulin are shown. *G–J*, shRNA knockdown of *Crebbp* in TS^WT^ cells using two independent *Crebbp* shRNAs (Csh1 and Csh2) modestly affects TRAF4 expression. *G,* qPCR data normalized to *Rps11* are expressed as a fold-change relative to control (C) TS^WT^ cells and are the mean ± SD of four biologically independent experiments. *H*, representative Western blots and (*I* and *J*) densitometry analyses are shown. *K–M*, *Hdac6* shRNA knockdown in TS^KI^ cells (H6sh) does not affect *Traf4* transcript but decreases TRAF4 protein levels. *K*, qPCR data normalized as in (*G*) show the mean ± SD of four biologically independent experiments. *L,* representative Western blots and (*M*) densitometry analyses are shown. *B*, *D*, *H*, and *L*, Western blots are representative of three biologically independent experiments. *C*, *E*, *F*, *I*, *J*, and *M*, bar graphs show the mean ± SD of three biologically independent experiments. ∗ *p*-value < 0.05; ∗∗ *p*-value < 0.01; ∗∗∗ *p*-value < 0.001; ∗∗∗∗ *p*-value < 0.0001; Student’s *t* test; C, control; JNK, c-Jun N-terminal kinase; ns, not significant; PI3K, phosphoinositide 3-kinase; qPCR, quantitative PCR; RPKM, reads per kilobase million.

Based on the coexpression of *Traf4* and *Map3k4* in mouse TS cells and the previously defined role of TRAF4 as an activator and binding partner of MAP3K4, we examined *Traf4* in TS^KI^ cells. Both *Traf4* transcript, measured by RNA-Seq, and TRAF4 protein, assessed by Western blotting, were significantly elevated in TS^KI^ cells relative to WT TS (TS^WT^) cells, suggesting a potential compensatory mechanism to overcome the loss of MAP3K4 activity (Fig. 2, *A*–*C*) (8, 10). MAP3K4 promotes the activity of the PI3K/Akt, p38, and JNK pathways in TS cells, and TS^KI^ cells exhibit diminished activation of these pathways (5, 7, 9). To determine the impact of reduced activation of these pathways on TRAF4, we treated TS^WT^ cells with pharmacological inhibitors, observing statistically significant increases in TRAF4 protein expression in TS^WT^ cells individually treated with inhibitors for PI3K, p38, or JNK relative to control TS^WT^ cells (Fig. 2, *D* and *E*). The increased expression of TRAF4 in TS cells upon inhibition of MAP3K4 activity–dependent pathways is consistent with TRAF4 upregulation in TS^KI^ cells and further supports a possible role for TRAF4 in attempting to compensate for MAP3K4 inactivation. We similarly observed upregulation of HDAC6 upon inhibition of these pathways, consistent with HDAC6 expression in TS cells being MAP3K4-dependent (Fig. 2, *D* and *F*) (8). TRAF4 and HDAC6 are each members of large protein families. To identify potential compensatory roles of other TRAFs and HDACs in the TS cells, we examined TS^WT^ and TS^KI^ cell RNA-Seq data (8, 10). Our data indicated that of the Trafs reaching an expression threshold of 1 RPKM, *Traf4* is the only Traf with increased expression in TS^KI^ cells (Table S1). In addition to increased *Hdac6* expression, *Hdac7* was also increased in TS^KI^ cells (Table S1). These findings suggest that though other TRAFs or HDACs may play roles upstream or downstream of MAP3K4, TRAF4 and HDAC6 are the family members most upregulated upon MAP3K4 kinase inactivation.

We have previously shown that MAP3K4 promotes the activity of the histone acetyltransferase CREB-binding protein (CBP) and inhibits the expression and activity of HDAC6 (6, 8). This MAP3K4, CBP, and HDAC6-dependent regulatory network led us to hypothesize that the upregulation of *Traf4* in TS^KI^ cells may be due in part to loss of MAP3K4-dependent regulation of CBP or HDAC6. We examined the impact of CBP shRNA knockdown in TS^WT^ cells on *Traf4* expression. *Traf4* transcript levels, measured by quantitative PCR (qPCR), were unaffected by CBP knockdown compared to control TS^WT^ cells (Fig. 2*G*). TRAF4 protein levels were modestly decreased but required near complete knockdown of CBP (Fig. 2, *H*–*J*). Since CBP activity is decreased in TS^KI^ cells, it is unlikely that increased TRAF4 in TS^KI^ cells is CBP-dependent (6). TS^KI^ cells exhibit increased HDAC6 expression and activity compared to TS^WT^ cells due in part to disrupted HDAC6 degradation (8). Knockdown of *Hdac6* in TS^KI^ cells did not affect *Traf4* transcript relative to control TS^KI^ cells as measured by qPCR (Fig. 2*K*). However, *Hdac6* shRNA knockdown in TS^KI^ cells decreased TRAF4 protein expression to levels similar to TS^WT^ cells (Fig. 2, *L* and *M*). The lack of effect of HDAC6 on *Traf4* transcript and the significant impact of HDAC6 on TRAF4 protein expression suggested that HDAC6 may regulate TRAF4 expression through posttranslational mechanisms.

### HDAC6 and TRAF4 form a complex that stabilizes TRAF4 expression

**Figure 3:**
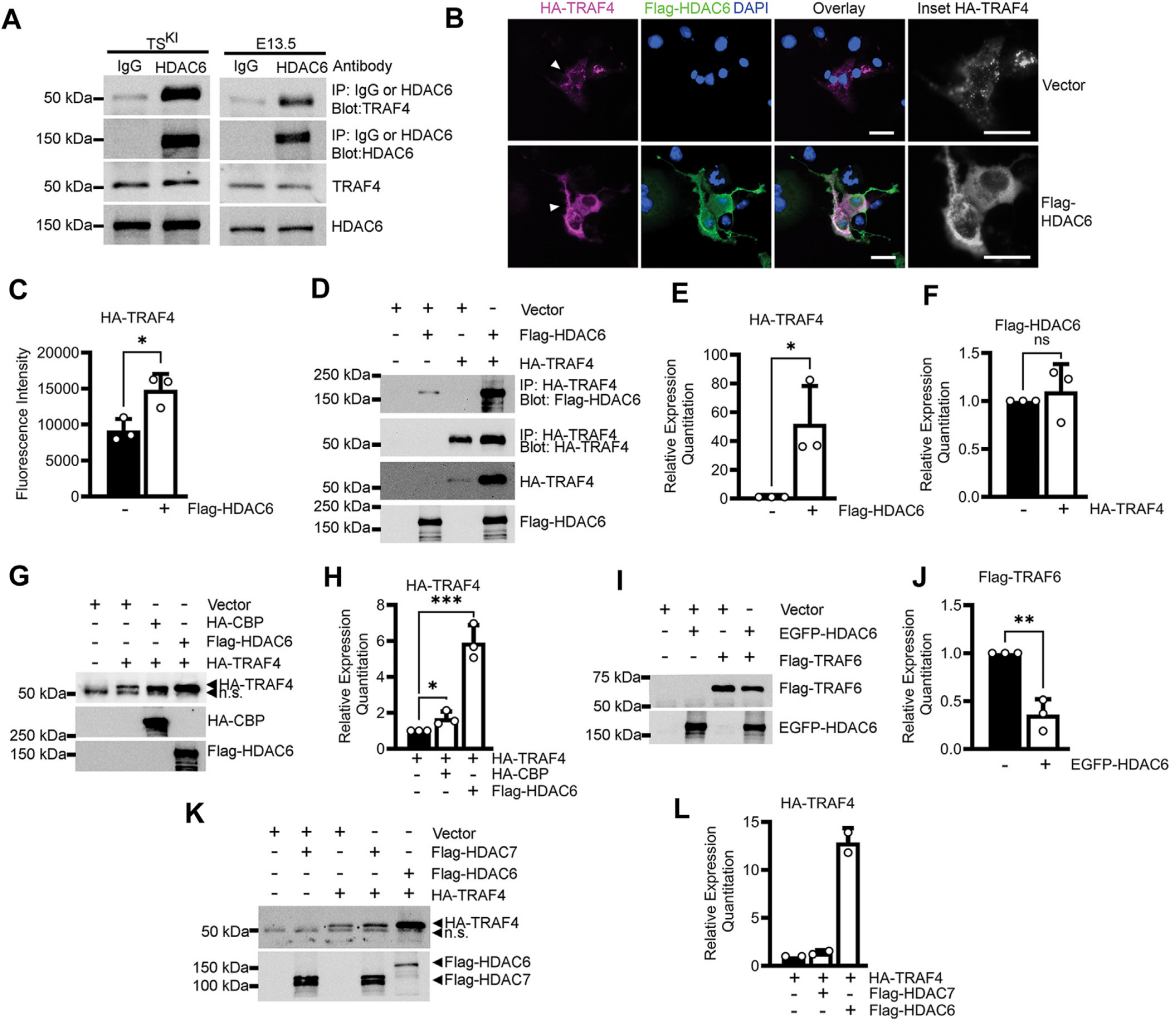
**HDAC6 forms a complex with TRAF4 that promotes TRAF4 expression.***A*, endogenous coimmunoprecipitation of TRAF4 with HDAC6 in TS^KI^ cells and E13.5 embryos. Representative Western blots are shown. *B* and *C*, colocalization of HA-TRAF4 and Flag-HDAC6 increases HA-TRAF4 expression. *B*, COS-7 immunofluorescence images; HA (*magenta*), Flag (*green*), and DAPI nuclei (*blue*). *Arrowhead* indicates inset location. Scale bar represents 20 μm. *C*, quantitation of HA-TRAF4 fluorescence intensity from *B* are the mean ± SD of three biologically independent experiments. Each dot represents the mean fluorescence intensity of a minimum of 15 regions of interest from one biologically independent experiment. *D–L,* HEK293T transfection experiments. *D–F,* Flag-HDAC6 coimmunoprecipitates with HA-TRAF4 and increases HA-TRAF4 expression. *D*, representative Western blots and (*E*) densitometry analyses of HA-TRAF4 normalized to samples without Flag-HDAC6 or (*F*) Flag-HDAC6 normalized to samples without HA-TRAF4. *G* and *H*, CBP overexpression modestly increases TRAF4 expression. *G*, representative Western blots and (*H*) densitometry analyses of HA-TRAF4 normalized to samples without HA-CBP or Flag-HDAC6. *I* and *J*, HDAC6 does not stabilize TRAF6 expression. *I*, representative Western blots and (*J*) densitometry analyses of Flag-TRAF6 normalized to samples without EGFP-HDAC6. *K* and *L*, HDAC7 does not affect TRAF4 expression. *K*, representative Western blots. *L*, densitometry analyses of HA-TRAF4 normalized to samples without Flag-HDACs. Bar graphs show the mean ± range of two biologically independent experiments. *A*, *D*, *G*, and *I*, Western blots are representative of three biologically independent experiments. *C*, *E*, *F*, *H,* and *J*, bar graphs show the mean ± SD of three biologically independent experiments. ∗ *p*-value < 0.05; ∗∗ *p*-value < 0.01; ∗∗∗ *p*-value < 0.001; Student’s *t* test; ns, not significant; n.s., nonspecific.

Based on the impact of HDAC6 on TRAF4 protein levels, we wondered if HDAC6 and TRAF4 formed a protein complex. TRAF4 and HDAC6 coimmunoprecipitated in TS^KI^ cells and E13.5 embryo lysates, showing endogenous complex formation (Fig. 3*A*). To examine the localization of the TRAF4 and HDAC6 complex, we performed immunostaining in transfected COS-7 cells. HA-TRAF4 was localized in small cytoplasmic puncta in the absence of Flag-HDAC6 (Fig. 3*B*). This punctate localization was consistent with published overexpression of HA-TRAF4 in COS-7 cells and Flag-TRAF4 in various cell lines (11, 29). When coexpressed, HA-TRAF4 and Flag-HDAC6 colocalized in the cytoplasm of COS-7 cells, and HA-TRAF4 distribution appeared more diffuse (Fig. 3*B*). Further, HA-TRAF4 expression appeared to increase when coexpressed with Flag-HDAC6 (Fig. 3*B*). Quantitation showed that this increase was statistically significant (Fig. 3*C*). Coexpressed HA-TRAF4 and Flag-HDAC6 coimmunoprecipitated in HEK293T cells (Fig. 3*D*). Significantly, coexpression resulted in a robust and statistically significant increase in HA-TRAF4 expression but had no effect on Flag-HDAC6 expression (Fig. 3, *D*–*F*). In contrast, while coexpression with Flag-HDAC6 robustly increased HA-TRAF4 expression, coexpression with HA-CBP resulted in a modest 1.7-fold increase in HA-TRAF4 (Fig. 3, *G* and *H*). HDAC6 failed to stabilize TRAF6, and HDAC7 did not affect the expression of TRAF4 (Fig. 3, *I*–*L*). Taken together, our data demonstrate that HDAC6 forms a complex with TRAF4 and promotes TRAF4 stability.

### HDAC6 binds and stabilizes the TRAF domain of TRAF4

**Figure 4:**
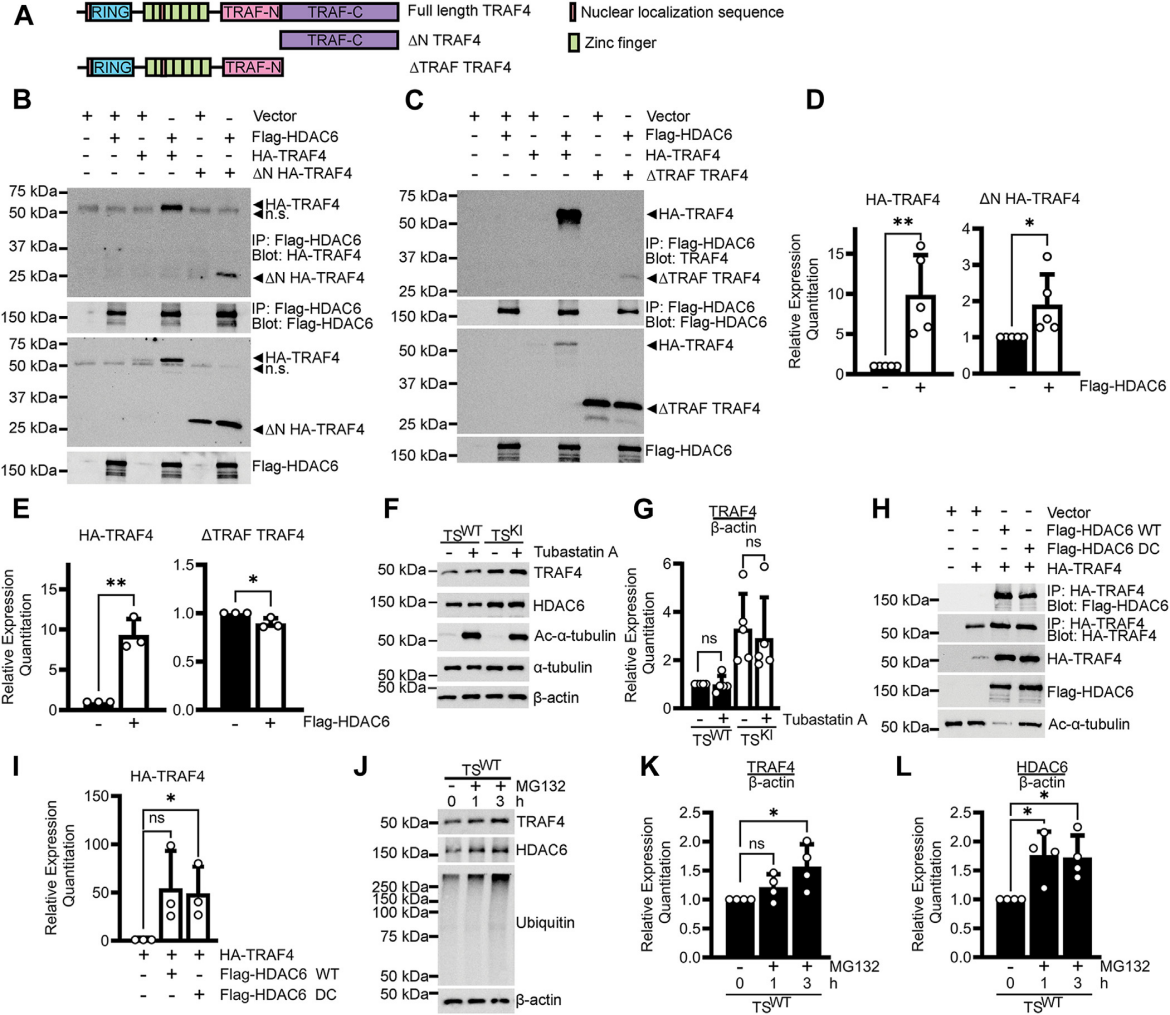
**Interaction between HDAC6 and the TRAF domain of TRAF4 does not require HDAC6 deacetylase activity.***A*, protein domain maps of TRAF4 deletion mutants. *B*, the TRAF4 TRAF domain coimmunoprecipitates with Flag-HDAC6. Western blots of coimmunoprecipitations are representative of three biologically independent experiments. *C*, weak coimmunoprecipitation of the TRAF4 N-terminus with Flag-HDAC6. Western blots of coimmunoprecipitations are representative of two biologically independent experiments. *D*, densitometry analyses of total levels of full-length TRAF4 and TRAF4 TRAF domain from *B* are normalized to samples without Flag-HDAC6 and show the mean ± SD of five biologically independent experiments. *E*, densitometry analyses of total levels of full-length TRAF4 and TRAF4 N-terminus from *C* are normalized to samples without Flag-HDAC6 and show the mean ± SD of three biologically independent experiments. *F* and *G*, inhibition of HDAC6 in TS cells does not impact TRAF4 protein expression. TS cells were treated with either DMSO or 10 μM tubastatin A for 48 h. *F*, representative Western blots. *G*, densitometry analyses show the mean ± SD of five biologically independent experiments. *H* and *I*, TRAF4 stabilization and complex formation by HDAC6 occurs in the absence of HDAC6 deacetylase activity. Deacetylase-deficient Flag-HDAC6 (Flag-HDAC6 DC) binds and stabilizes TRAF4. *H*, representative Western blots. *I*, densitometry analyses of HA-TRAF4 normalized to samples without Flag-HDAC6 show the mean ± SD of three biologically independent experiments. *J–L*, inhibition of the proteasome in TS^WT^ cells increases TRAF4 and HDAC6 expression. TS^WT^ cells were treated with DMSO or 10 μM MG132 for the indicated number of hours. *J*, representative Western blots. *K* and *L*, densitometry analyses show the mean ± SD of four biologically independent experiments. *B–E*, *H*, and *I*, HEK293T transfection experiments. ∗ *p*-value < 0.05; ∗∗ *p*-value < 0.01; Student’s *t* test; one-way ANOVA with Dunnett’s *post hoc* test; ns, not significant; n.s., nonspecific.

To further define HDAC6–TRAF4 interactions, we examined HDAC6 complex formation with either the TRAF domain alone (ΔN HA-TRAF4) or the N-terminus (ΔTRAF TRAF4) (Fig. 4*A*). Flag-HDAC6 coimmunoprecipitated with the TRAF domain alone but exhibited notably diminished binding to the N-terminus lacking the TRAF domain (compare Fig. 4*B* and *C*). We wondered how the expression of the TRAF domain alone or the N-terminus alone changed with coexpression with Flag-HDAC6. Additional experiments examining total protein levels of the TRAF4 constructs demonstrated that only proteins containing the TRAF domain were increased with HDAC6 coexpression (compare Fig. 4, *B*–*E*). Taken together, these data demonstrate that the TRAF domain of TRAF4 is both stabilized by and sufficient for complex formation with HDAC6.

### Complex formation with TRAF4 can occur in the absence of HDAC6 deacetylase activity

HDAC6 regulates the deacetylation of substrates like α-tubulin and cortactin through two N-terminal deacetylase domains (30, 31). To determine if HDAC6 regulates TRAF4 expression through deacetylation, we examined the impact of HDAC6 inhibition on TRAF4 expression in TS cells using the HDAC6-selective inhibitor tubastatin A (32). There was no change in TRAF4 expression upon treatment with tubastatin A compared to the vehicle controls, suggesting that HDAC6 regulation of TRAF4 expression may be independent of HDAC6 enzymatic activity (Fig. 4, *F* and *G*). Based on these data, we compared HA–TRAF4 complex formation with Flag-HDAC6 WT or a deacetylase-deficient Flag-HDAC6 (Flag-HDAC6 DC). The absence of deacetylase activity of the Flag-HDAC6 DC mutant was confirmed by acetylation of α-tubulin (Fig. 4*H*). Both Flag-HDAC6 WT and Flag-HDAC6 DC coimmunoprecipitated with HA-TRAF4 and increased HA-TRAF4 expression to similar levels, indicating that complex formation and stabilization of TRAF4 can occur independent of HDAC6 deacetylase activity (Fig. 4, *H* and *I*). Previous studies have demonstrated roles for HDAC6 in binding ubiquitinated proteins and regulating ubiquitin chain turnover (33). To determine potential deacetylase-independent functions of HDAC6 on TRAF4, we examined the impact of proteasome or lysosome inhibition on HDAC6 and TRAF4 expression. Although inhibition of the lysosome did not impact TRAF4 or HDAC6 levels, inhibition of the proteasome with MG132 resulted in the accumulation of ubiquitin and increased TRAF4 and HDAC6 expression, suggesting both TRAF4 and HDAC6 are targeted by the proteasome (Figs. S3 and 4, *J–L*). The accumulation of TRAF4 and HDAC6 upon proteasome inhibition and our data displaying TRAF4 binding and stabilization by deacetylase-deficient HDAC6 supports a potential deacetylase-independent function for HDAC6 in regulating TRAF4 degradation by the proteasome. Taken together, our data indicate that HDAC6 interacts with the TRAF domain of TRAF4 and stabilizes TRAF4 in a manner that does not require deacetylase activity.

### Coexpression of Traf4 with Map3k4 or Hdac6 in placental trophoblasts changes with differentiation

**Figure 5:**
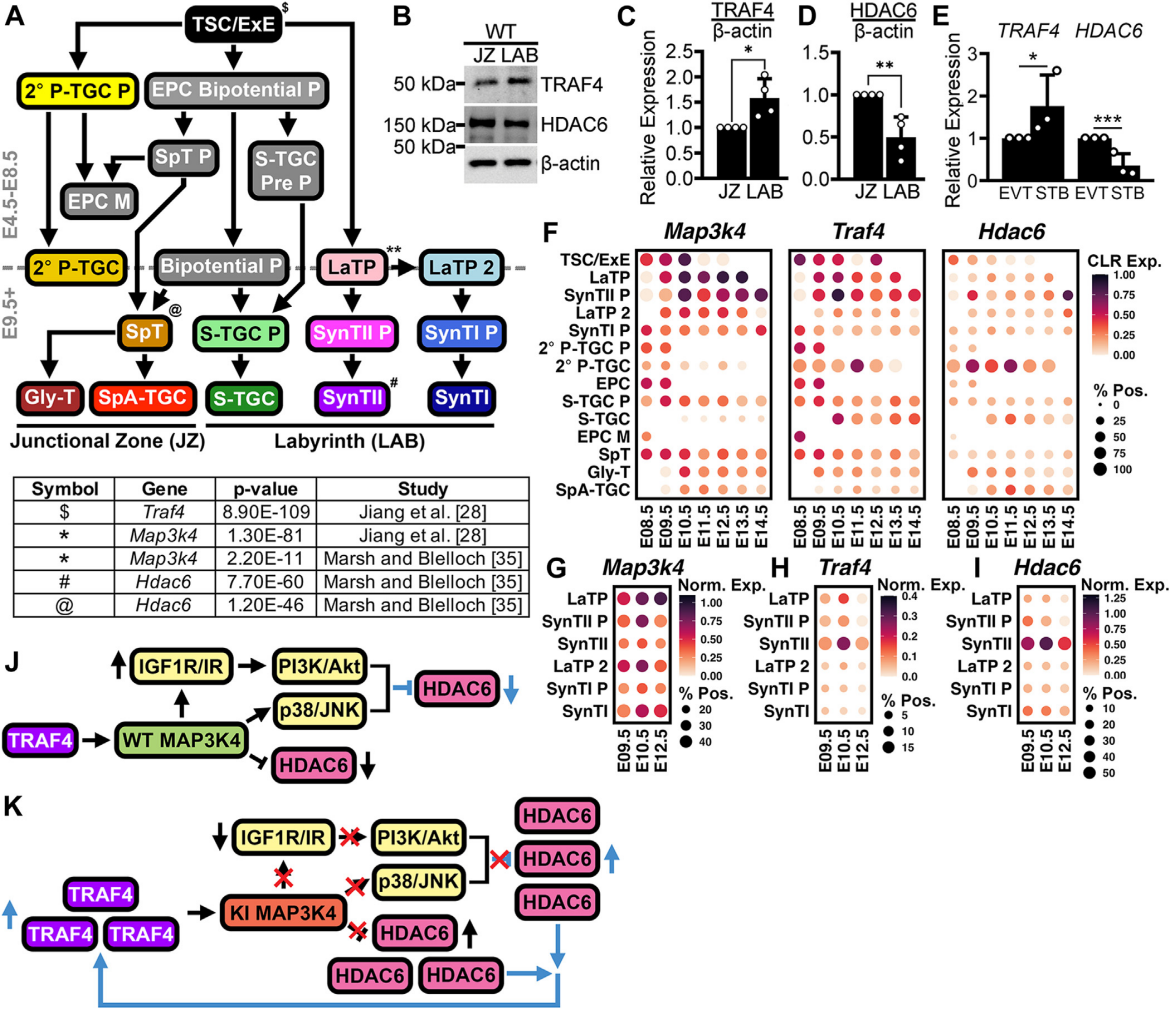
**Coexpression of *Traf4* with *Map3k4* or *Hdac6* changes with trophoblast differentiation during development.***A*, flow chart of trophoblast differentiation during placental development adapted from Jiang *et al.* (2023). Cell types for which *Traf4*, *Map3k4*, or *Hdac6* are markers are indicated by symbols with corresponding *p*-values. *B–D*, TRAF4 and HDAC6 are differentially expressed in TS cells differentiated *in vitro* to the JZ or LAB. *B*, representative Western blots. *C* and *D*, densitometry analyses normalized to expression in the JZ are the mean ± SD of four biologically independent experiments. *E*, *TRAF4* and *HDAC6* are differentially expressed between human TS cells differentiated *in vitro* to EVT or STB. Bar graphs show the mean ± SD of three biologically independent experiments normalized to expression in the EVT. *F*, dot plots of scRNAseq data show differences in *Map3k4*, *Traf4*, and *Hdac6* expression in placental trophoblasts. *G–I*, dot plots of snRNAseq data show differences in *Map3k4*, *Traf4*, and *Hdac6* expression in LAB cells. *J* and *K*, MAP3K4-dependent regulation of TRAF4 and HDAC6 in (*J*) TS^WT^ and (*K*) TS^KI^ cells. *J*, MAP3K4 promotes the activation of the p38 and JNK MAPKs, and IGF1R/IR and PI3K/Akt pathways. TRAF4 binds MAP3K4 and promotes MAP3K4 homodimerization and activation, whereas interactions of WT MAP3K4 with HDAC6 promote HDAC6 degradation, and activation of MAP3K4-dependent pathways inhibits HDAC6 expression. *K*, MAP3K4 kinase inactivation results in reduced activation of MAP3K4-dependent pathways. KI MAP3K4 binds TRAF4 and HDAC6 but results in increased HDAC6 expression due in part to disruption of MAP3K4-dependent pathways and HDAC6 degradation. Overexpression of HDAC6 promotes HDAC6 binding and stabilization of TRAF4, resulting in TRAF4 overexpression in TS^KI^ cells. *J* and *K*, *black arrows* indicate previously published findings. *Blue arrows* indicate findings from this study. ∗ *p*-value < 0.05; ∗∗ *p*-value < 0.01; ∗∗∗ *p*-value < 0.001; Student’s *t* test; Akt, protein kinase B; Bipotential P, bipotential progenitor; Exp, expression; IGF1R, insulin-like growth factor 1 receptor; IR, insulin receptor; JNK, c-Jun N-terminal kinase; JZ, junctional zone; KI, kinase-inactive; LAB, labyrinth; MAPK, mitogen-activated protein kinase; Norm, normalized; PI3K, phosphoinositide 3-kinase; Pos, positive; scRNAseq, single-cell RNAseq; snRNAseq, single-nuclei RNAseq; SynTI, SynT layer I; SynTII, SynT layer II; (see Fig. 1 legend for additional abbreviations).

Our mechanistic investigations of the regulation of TRAF4 by MAP3K4 and HDAC6 in TS cells led us to wonder about their potential relationships in differentiated trophoblasts. Figure 5*A* displays a flow chart of placental trophoblast differentiation adapted from Jiang *et al.* (28). During development, TS/Extraembryonic Ectoderm (ExE) cells differentiate to form multiple trophoblast progenitor cell types, which further differentiate to form the mature cells of the junctional zone (JZ) or labyrinth (LAB). Though we have shown that TRAF4 is abundant in TS cells, we wondered about potential roles for TRAF4 and its relationships with MAP3K4 and HDAC6 in JZ or LAB cell lineages. We performed *in vitro* differentiation of TS^WT^ cells into the JZ or LAB and saw TRAF4 expression was significantly greater in LAB cells than the JZ (Fig. 5, *B* and *C*). In contrast, HDAC6 expression was lower in LAB cells compared to the JZ (Fig. 5, *B* and *D*). Likewise, analyses of *in vitro* differentiation of human trophoblasts showed *TRAF4* expression was higher in syncytiotrophoblast cells with similarities to the mouse LAB than the extravillous trophoblasts with similarities to cells of the JZ (Fig. 5*E*) (34). *HDAC6* expression was higher in the extravillous trophoblasts than syncytiotrophoblasts (Fig. 5*E*) (34). We wondered how *in vitro* trends compared to the changes *in vivo* during development. We examined the average centered-log ratio expression of *Map3k4*, *Traf4*, and *Hdac6* in differentiated trophoblasts during development using the scRNAseq data set (Fig. 5*F*) (28). We observed abundant *Map3k4* and *Traf4* expression in TS/ExE cells, LAB trophoblast progenitor (LaTP) cells, and the syncytiotrophoblast layer II precursor (SynTII P) cells that form from the LaTPs (Fig. 5*F*). Moreover, *Traf4* expression was more abundant in the LAB cell types than cells of the JZ, consistent with *in vitro* differentiation (Fig. 5, *B–F*). Intriguingly, *Hdac6* was expressed in both the JZ and specific LAB cells, particularly the SynTII P cells (Fig. 5*F*). We wondered if *Map3k4, Traf4,* and/or *Hdac6* were coexpressed in the fully differentiated SynTII cells, which are formed from the SynTII Ps (Fig. 5*A*). However, due to the limitations of scRNAseq, the fully differentiated, multinucleated SynTII cells could not be captured for sequencing, causing these cell types to be absent (28). To bypass these issues, we used a single-nuclei RNA-Seq data set of the mouse placental LAB, which was able to capture the missing cell types (35). Consistent with the scRNAseq data, we saw abundant coexpression of *Map3k4* and *Traf4* in LaTP and SynTII P cells; however, expression of *Map3k4* decreased with differentiation to the SynTII cells (Fig. 5, *G* and *H*). In contrast, *Traf4* and *Hdac6* coexpression was highest in the SynTIIs, suggesting a potential role for TRAF4 and HDAC6 in the SynTII cells (Fig. 5, *H* and *I*). Data mining of trophoblast subtype clusters from Jiang *et al.* and Marsh and Blelloch allowed us to further validate the enrichment of *Map3k4* in the LaTPs, *Traf4* in TS cells, and *Hdac6* in spongiotrophoblasts and SynTIIs with supporting *p*-values (Fig. 5*A*) (28, 35). Taken together, these data highlight previously unknown mechanisms of MAP3K4-dependent regulation of HDAC6 and TRAF4 in TS cells and indicate a switch in coexpression between *Map3k4*, *Traf4*, and *Hdac6* in differentiated trophoblasts during placental development. In TS^WT^ cells, MAP3K4 and TRAF4 are coexpressed, and MAP3K4 negatively regulates HDAC6 expression through activation of signaling pathways and promoting HDAC6 degradation (Fig. 5*J*). However, kinase inactivation of MAP3K4 in TS cells leads to changes in HDAC6 and TRAF4 expression levels, as HDAC6 overexpression results in increased TRAF4 expression (Fig. 5*K*). These alterations in MAP3K4, TRAF4, and HDAC6 coexpression also occur during trophoblast differentiation, suggesting these relationships and regulatory mechanisms may play important roles in placental development. Together, our study demonstrates a previously unknown role of HDAC6 as a binding partner and positive regulator of TRAF4 expression and examines MAP3K4, TRAF4, and HDAC6 associations during differentiation of placental trophoblasts.

## Discussion

Herein, our findings demonstrate previously unknown associations of TRAF4 with MAP3K4 and HDAC6 in TS cells and differentiated placental trophoblasts. TRAF4 and MAP3K4 are abundantly expressed in TS cells and LAB progenitors, whereas HDAC6 expression is higher in differentiated trophoblasts. Loss of MAP3K4 activity and inhibition of MAP3K4-dependent pathways in TS cells increase TRAF4 and HDAC6 expression. We define a molecular mechanism by which overexpression of HDAC6 due to loss of MAP3K4 activity promotes TRAF4 expression. We demonstrate that HDAC6 forms a complex with and stabilizes TRAF4, and these effects can occur in the absence of HDAC6 deacetylase activity. Importantly, we examine these relationships among MAP3K4, TRAF4, and HDAC6 in TS cells and differentiated trophoblasts and show differential coexpression of TRAF4 with MAP3K4 *versus* HDAC6 in cells of the LAB. Overall, these data suggest potential roles for MAP3K4, TRAF4, and HDAC6 associations in placental development that may be disrupted by MAP3K4 kinase inactivation and promote MAP3K4 KI phenotypes.

MAP3K4 promotes the activation of the p38 and JNK MAPKs and the insulin-like growth factor 1 receptor (IGF1R), insulin receptor, and PI3K/Akt signaling pathways in TS cells (5, 6, 7, 8, 9). As a MAP3K4-binding partner, TRAF4 binds the kinase domain of MAP3K4, enhancing MAP3K4 homodimerization and activation of downstream pathways (11). HDAC6 binds the kinase domain of MAP3K4, and MAP3K4 interactions with HDAC6 promote HDAC6 ubiquitination and degradation (8). We have previously shown a role for MAP3K4 in regulating fetal and placental growth, where MAP3K4 activation of CBP and inhibition of HDAC6 promotes transcript expression of *Igf1r* and activation of the IGF1R/IR and PI3K/Akt pathways (9). Our new study expands our understating of these relationships among MAP3K4, TRAF4, and HDAC6 in the placenta, highlighting previously unknown functions of MAP3K4 expression and signaling in regulating HDAC6 and TRAF4 in TS cells and differentiated trophoblasts. We found that TRAF4 and MAP3K4 are abundantly expressed in TS cells and LAB progenitors, and expression of MAP3K4 and TRAF4 decreases with trophoblast differentiation. In contrast, HDAC6 expression is lower in TS cells and enriched in differentiated trophoblasts. Inhibition of PI3K/Akt, p38, or JNK pathways in TS^WT^ cells results in modest but significant increases in HDAC6 expression, consistent with its MAP3K4 dependence. Moreover, TRAF4 expression increases with inhibition of MAP3K4-dependent pathways in TS^WT^ cells. These alterations in HDAC6 and TRAF4 expression may represent mechanisms of attempted compensation for the loss of MAP3K4 activity to drive activation of MAP3K4 pathways in TS cells. Moreover, increases in HDAC6 and TRAF4 upon inhibition of MAP3K4-dependent pathways may explain changes in HDAC6 and TRAF4 expression that occur with loss of MAP3K4 during differentiation. As *Map3k4* levels decrease during normal differentiation of the LaTP lineage, the expression of *Traf4* and *Hdac6* are increased in the SynTIIs. These changes in expression further support that HDAC6 expression is increased with loss of MAP3K4 and suggest that HDAC6 may play a role in promoting TRAF4 expression during LAB differentiation. The LAB is the zone of the placenta where the exchange of gases, nutrients, and waste between the mother and fetus occurs, whereas the JZ provides hormonal and structural support to the placenta and invades the maternal decidua. Changes in expression suggest potential roles for cell-type–specific MAP3K4, TRAF4, and HDAC6 relationships in placental LAB formation and function during normal development.

While our findings suggest potential normal roles for MAP3K4, TRAF4, and HDAC6 in placental development, dysregulation and overexpression of TRAF4 and HDAC6 may function in the pathologies of *Map3k4*^*KI/KI*^ placentas. We have previously shown MAP3K4 kinase inactivation promotes trophoblast hyperinvasion and placental insufficiency due in part to dysregulation of HDAC6 (6, 8, 9). Our new study shows that HDAC6 binds TRAF4 in TS^KI^ cells, and interactions of TRAF4 with HDAC6 robustly increase TRAF4 expression. Dysregulated TRAF4 expression is correlated with disease states including cancer and inflammation, both of which are promoted by HDAC6 (19, 36, 37). Other studies have shown that the TRAF domain of TRAF4 binds Arg-Leu-X-Ala (RLXA) motifs in other proteins (16). Intriguingly, this motif is present in catalytic domain 1 of HDAC6 (RLHA) and is conserved in both human and mouse HDAC6. The binding of HDAC6 to the TRAF4 TRAF domain combined with strong, deacetylase-independent effects of HDAC6 on TRAF4 protein expression are consistent with a role for HDAC6 in protecting TRAF4 from interactions that lead to TRAF4 degradation. This role for HDAC6 is supported by its defined roles in binding and trafficking of ubiquitinated proteins, promoting aggresome assembly, and delaying processing of ubiquitinated proteins by the ubiquitin-proteasome system (33, 38, 39). The overexpression of HDAC6 and the protective role of HDAC6 on TRAF4, resulting in the overexpression of TRAF4 in TS^KI^ cells, may function in promoting TS^KI^ cell phenotypes and placental defects *in vivo*.

Overexpression of TRAF4 and HDAC6 may also affect LAB formation in *Map3k4*^*KI/KI*^ placentas. *In vitro* TS^KI^ cell differentiation to SynTIIs is disrupted, consistent with a role for MAP3K4 in the differentiation to this cell type (9). *Map3k4*^*KI/KI*^ placentas show decreased placental size and weight characteristic of placental insufficiency, likely due in part to impaired differentiation of TS^KI^ cells to SynTIIs (9). This disruption of LAB formation upon MAP3K4 kinase inactivation may reflect impaired LAB trophoblast differentiation, in which cells may become trapped in a stem cell or progenitor state or preferentially differentiate to JZ lineages. The abundance of *Traf4 in vivo* in placental TS cells and the overexpression of TRAF4 and HDAC6 in TS^KI^ cells may implicate TRAF4 and HDAC6 in maintaining TS cell stemness upon loss of MAP3K4 activity and may impede LAB differentiation. However, HDAC6 overexpression may also selectively promote differentiation to spongiotrophoblasts of the JZ instead of the SynTIIs of the LAB, consistent with *Hdac6* enrichment in these cells. We hypothesize that dysregulation of TRAF4 and HDAC6 due in part to loss of MAP3K4 activity may function in the disruption of LAB differentiation and formation in *Map3k4*^*KI/KI*^ placentas.

In summary, our study demonstrates TRAF4 is abundant in TS cells of the placenta, and HDAC6 and MAP3K4 coregulate TRAF4 expression in TS cells. HDAC6 binds and stabilizes TRAF4, and MAP3K4, TRAF4, and HDAC6 are differentially coexpressed during placental differentiation. Our work demonstrates previously unknown relationships and interactions between TRAF4 and HDAC6 in normal placental development. Future studies will examine TRAF4 and HDAC6 functions *in vivo* in *Map3k4*^*KI/KI*^ differentiated trophoblasts. Investigations of these functions of HDAC6 and TRAF4 in *Map3k4*^*KI/KI*^ placentas may present HDAC6 and TRAF4 as potential therapeutic targets for interventions to improve placental development.

## Experimental procedures

All experiments using animals were approved by the Institutional Animal Care and Use Committee (IACUC) at the University of Memphis. All experiments using animals were performed according to institutional and National Institutes of Health guidelines and regulations. Detailed information about experimental procedures is included in the Supporting information.

## Data availability

The authors declare that all data underlying this work are present in the published paper.

## Supporting information

This article contains Supporting information (5, 6, 8, 9, 10, 12, 40, 41, 42, 43, 44, 45, 46, 47, 48).

## Conflict of interest

The authors declare that they have no conflicts of interest with the contents of this article.

## References

[1] Brett K.E., Ferraro Z.M., Yockell-Lelievre J., Gruslin A., Adamo K.B. (2014). Maternal-fetal nutrient transport in pregnancy pathologies: the role of the placenta. Int. J. Mol. Sci..

[2] Burton G.J., Jauniaux E. (2018). Pathophysiology of placental-derived fetal growth restriction. Am. J. Obstet. Gynecol..

[3] Woods L., Perez-Garcia V., Hemberger M. (2018). Regulation of placental development and its impact on fetal growth-new insights from mouse models. Front. Endocrinol. (Lausanne).

[4] Tanaka S., Kunath T., Hadjantonakis A.K., Nagy A., Rossant J. (1998). Promotion of trophoblast stem cell proliferation by FGF4. Science.

[5] Abell A.N., Granger D.A., Johnson N.L., Vincent-Jordan N., Dibble C.F., Johnson G.L. (2009). Trophoblast stem cell maintenance by fibroblast growth factor 4 requires MEKK4 activation of Jun N-terminal kinase. Mol. Cell Biol..

[6] Abell A.N., Jordan N.V., Huang W., Prat A., Midland A.A., Johnson N.L. (2011). MAP3K4/CBP-regulated H2B acetylation controls epithelial-mesenchymal transition in trophoblast stem cells. Cell Stem Cell.

[7] Abell A.N., Rivera-Perez J.A., Cuevas B.D., Uhlik M.T., Sather S., Johnson N.L. (2005). Ablation of MEKK4 kinase activity causes neurulation and skeletal patterning defects in the mouse embryo. Mol. Cell Biol..

[8] Mobley R.J., Raghu D., Duke L.D., Abell-Hart K., Zawistowski J.S., Lutz K. (2017). MAP3K4 controls the chromatin modifier HDAC6 during trophoblast stem cell epithelial-to-mesenchymal transition. Cell Rep..

[9] Perry C.H., Mullins N.A., Sweileh R.B.A., Shendy N.A.M., Roberto P.A., Broadhurst A.L. (2022). MAP3K4 promotes fetal and placental growth by controlling the receptor tyrosine kinases IGF1R/IR and Akt signaling pathway. J. Biol. Chem..

[10] Shendy N.A.M., Raghu D., Roy S., Perry C.H., Safi A., Branco M.R. (2020). Coordinated regulation of Rel expression by MAP3K4, CBP, and HDAC6 controls phenotypic switching. Commun. Biol..

[11] Abell A.N., Johnson G.L. (2005). MEKK4 is an effector of the embryonic TRAF4 for JNK activation. J. Biol. Chem..

[12] Sax J.K., El-Deiry W.S. (2003). Identification and characterization of the cytoplasmic protein TRAF4 as a p53-regulated proapoptotic gene. J. Biol. Chem..

[13] Wajant H., Henkler F., Scheurich P. (2001). The TNF-receptor-associated factor family: scaffold molecules for cytokine receptors, kinases and their regulators. Cell. Signal..

[14] Krajewska M., Krajewski S., Zapata J.M., Van Arsdale T., Gascoyne R.D., Berern K. (1998). TRAF-4 expression in epithelial progenitor cells. Analysis in normal adult, fetal, and tumor tissues. Am. J. Pathol..

[15] Esparza E.M., Arch R.H. (2004). TRAF4 functions as an intermediate of GITR-induced NF-kappaB activation. Cell Mol. Life Sci..

[16] Kim C.M., Son Y.J., Kim S., Kim S.Y., Park H.H. (2017). Molecular basis for unique specificity of human TRAF4 for platelets GPIbbeta and GPVI. Proc. Natl. Acad. Sci. U. S. A..

[17] Tomasetto C., Regnier C., Moog-Lutz C., Mattei M.G., Chenard M.P., Lidereau R. (1995). Identification of four novel human genes amplified and overexpressed in breast carcinoma and localized to the q11-q21.3 region of chromosome 17. Genomics.

[18] Regnier C.H., Tomasetto C., Moog-Lutz C., Chenard M.P., Wendling C., Basset P. (1995). Presence of a new conserved domain in CART1, a novel member of the tumor necrosis factor receptor-associated protein family, which is expressed in breast carcinoma. J. Biol. Chem..

[19] Camilleri-Broet S., Cremer I., Marmey B., Comperat E., Viguie F., Audouin J. (2007). TRAF4 overexpression is a common characteristic of human carcinomas. Oncogene.

[20] Liu K., Wu X., Zang X., Huang Z., Lin Z., Tan W. (2017). TRAF4 regulates migration, invasion, and epithelial-mesenchymal transition *via* PI3K/AKT signaling in hepatocellular carcinoma. Oncol. Res..

[21] Zhang J., Li X., Yang W., Jiang X., Li N. (2014). TRAF4 promotes tumorigenesis of breast cancer through activation of Akt. Oncol. Rep..

[22] Yang K., Wang F., Han J.J. (2015). TRAF4 promotes the growth and invasion of colon cancer through the Wnt/beta-catenin pathway. Int. J. Clin. Exp. Pathol..

[23] Yang J., Wei D., Wang W., Shen B., Xu S., Cao Y. (2015). TRAF4 enhances oral squamous cell carcinoma cell growth, invasion and migration by Wnt-beta-catenin signaling pathway. Int. J. Clin. Exp. Pathol..

[24] He S., Dong D., Lin J., Wu B., Nie X., Cai G. (2022). Overexpression of TRAF4 promotes lung cancer growth and EGFR-dependent phosphorylation of ERK5. FEBS Open Bio.

[25] Masson R., Regnier C.H., Chenard M.P., Wendling C., Mattei M.G., Tomasetto C. (1998). Tumor necrosis factor receptor associated factor 4 (TRAF4) expression pattern during mouse development. Mech. Dev..

[26] Regnier C.H., Masson R., Kedinger V., Textoris J., Stoll I., Chenard M.P. (2002). Impaired neural tube closure, axial skeleton malformations, and tracheal ring disruption in TRAF4-deficient mice. Proc. Natl. Acad. Sci. U. S. A..

[27] Shiels H., Li X., Schumacker P.T., Maltepe E., Padrid P.A., Sperling A. (2000). TRAF4 deficiency leads to tracheal malformation with resulting alterations in air flow to the lungs. Am. J. Pathol..

[28] Jiang X., Wang Y., Xiao Z., Yan L., Guo S., Wang Y. (2023). A differentiation roadmap of murine placentation at single-cell resolution. Cell Discov..

[29] Kedinger V., Alpy F., Baguet A., Polette M., Stoll I., Chenard M.P. (2008). Tumor necrosis factor receptor-associated factor 4 is a dynamic tight junction-related shuttle protein involved in epithelium homeostasis. PLoS One.

[30] Hubbert C., Guardiola A., Shao R., Kawaguchi Y., Ito A., Nixon A. (2002). HDAC6 is a microtubule-associated deacetylase. Nature.

[31] Zhang X., Yuan Z., Zhang Y., Yong S., Salas-Burgos A., Koomen J. (2007). HDAC6 modulates cell motility by altering the acetylation level of cortactin. Mol. Cell.

[32] Butler K.V., Kalin J., Brochier C., Vistoli G., Langley B., Kozikowski A.P. (2010). Rational design and simple chemistry yield a superior, neuroprotective HDAC6 inhibitor, tubastatin A. J. Am. Chem. Soc..

[33] Boyault C., Gilquin B., Zhang Y., Rybin V., Garman E., Meyer-Klaucke W. (2006). HDAC6-p97/VCP controlled polyubiquitin chain turnover. EMBO J..

[34] Wang L.J., Chen C.P., Lee Y.S., Ng P.S., Chang G.D., Pao Y.H. (2022). Functional antagonism between DeltaNp63alpha and GCM1 regulates human trophoblast stemness and differentiation. Nat. Commun..

[35] Marsh B., Blelloch R. (2020). Single nuclei RNA-seq of mouse placental labyrinth development. Elife.

[36] Shen J., Qiao Y., Ran Z., Wang T. (2013). Different activation of TRAF4 and TRAF6 in inflammatory bowel disease. Med. Inflamm..

[37] Zepp J.A., Wu L., Qian W., Ouyang W., Aronica M., Erzurum S. (2015). TRAF4-SMURF2-mediated DAZAP2 degradation is critical for IL-25 signaling and allergic airway inflammation. J. Immunol..

[38] Boyault C., Zhang Y., Fritah S., Caron C., Gilquin B., Kwon S.H. (2007). HDAC6 controls major cell response pathways to cytotoxic accumulation of protein aggregates. Genes Dev..

[39] Lee J.Y., Koga H., Kawaguchi Y., Tang W., Wong E., Gao Y.S. (2010). HDAC6 controls autophagosome maturation essential for ubiquitin-selective quality-control autophagy. EMBO J..

[40] Raghu D., Mobley R.J., Shendy N.A.M., Perry C.H., Abell A.N. (2019). GALNT3 maintains the epithelial state in trophoblast stem cells. Cell Rep..

[41] Chen C., Okayama H. (1987). High-efficiency transformation of mammalian cells by plasmid DNA. Mol. Cell Biol..

[42] Shendy N.A.M., Broadhurst A.L., Shoemaker K., Read R., Abell A.N. (2021). MAP3K4 kinase activity dependent control of mouse gonadal sex determination. Biol. Reprod..

[43] Gao Y.S., Hubbert C.C., Yao T.P. (2010). The microtubule-associated histone deacetylase 6 (HDAC6) regulates epidermal growth factor receptor (EGFR) endocytic trafficking and degradation. J. Biol. Chem..

[44] Bradney C., Hjelmeland M., Komatsu Y., Yoshida M., Yao T.P., Zhuang Y. (2003). Regulation of E2A activities by histone acetyltransferases in B lymphocyte development. J. Biol. Chem..

[45] Kawaguchi Y., Kovacs J.J., McLaurin A., Vance J.M., Ito A., Yao T.P. (2003). The deacetylase HDAC6 regulates aggresome formation and cell viability in response to misfolded protein stress. Cell.

[46] Fischle W., Emiliani S., Hendzel M.J., Nagase T., Nomura N., Voelter W. (1999). A new family of human histone deacetylases related to Saccharomyces cerevisiae HDA1p. J. Biol. Chem..

[47] Nakamura K., Kimple A.J., Siderovski D.P., Johnson G.L. (2010). PB1 domain interaction of p62/sequestosome 1 and MEKK3 regulates NF-kappaB activation. J. Biol. Chem..

[48] Ye X., Mehlen P., Rabizadeh S., VanArsdale T., Zhang H., Shin H. (1999). TRAF family proteins interact with the common neurotrophin receptor and modulate apoptosis induction. J. Biol. Chem..

