# Supplementary material for: MAP3K4 signaling regulates HDAC6 and TRAF4 coexpression and stabilization in trophoblast stem cells
Source: J Biol Chem. 2024 Dec 20;301(2):108116. doi: 10.1016/j.jbc.2024.108116 (PMC11787431; doi:10.1016/j.jbc.2024.108116)
Supplement: Supplemental Data [file mmc1.pdf]

MAP3K4 signaling regulates HDAC6 and TRAF4 coexpression and stabilization in trophoblast stem cells

Hannah A. Nelson[1], Nathan A. Mullins[1], and Amy N. Abell[1 *]

Supplemental experimental procedures

Table S1

Table S2

Table S3

Table S4

Figure S1

Figure S2

Figure S3

Supplemental references

Supplemental experimental procedures

Murine placental UMAP analysis and data visualization

Gene expression data from single-cell RNA-Seq of E8.5-E14.5 placentas (GSE156125) were processed using R (4.4.1) and R studio (2024.04.0+735). Normalization was performed across the E8.5-E14.5 datasets individually by using the centered log-ratio (CLR) method and the 'Seurat' R package (5.0.2). The uniform manifold approximation and projection (UMAP) technique was used to reduce the dimensions of each individual dataset for visualization of CLR normalized gene expression through the 'umap' R package (0.2.10.0). Normalized data were also used to calculate the mean expression and percent positive values shown in all dot plots. The 'ggplot2' (3.5.1) and 'patchwork' (1.2.0) R packages were used to generate scatterplots, dot plots, and figures. These packages were also used to display gene expression data from single-nuclei RNA-Seq of E9.5-E12.5 placentas (GSE152248).

Trophoblast stem (TS) cell RNA-Seq

Bulk RNA-Seq of TS^{WT} and TS^{KI} cells was performed as previously described (1, 2). The sequencing data sets can be found under accession numbers GSE92425 and GSE148496.

Cell lines and culture conditions

TS^{WT} and TS^{KI} cells were isolated from crosses of mice heterozygous for a targeted mutation in MAP3K4, rendering MAP3K4 kinase-inactive (3). TS cells were cultured as previously described (1-5). To induce differentiation, TS cells were cultured in the absence of mouse embryonic fibroblast conditioned media (MEF-CM), fibroblast growth factor 4 (FGF4), and heparin. *Crebbp* or *Hdac6* shRNA knockdown cells were

created as previously described (1, 5). For experiments to inhibit MAP3K4-dependent pathways, TS cells were treated for 24 h with either vehicle control (DMSO) or 200 nM ZSTK474 (MedChemExpress) for PI3K inhibition, 10 μM BIRB796 (Tocris) for p38 inhibition, or 10 μM SP600125 (Sigma-Aldrich) for JNK inhibition. For HDAC6 inhibition, TS cells were treated with either 10 μM Tubastatin A (Selleck Chemicals) or DMSO every 24 h for 48 h. For inhibition of the proteasome, TS^{WT} cells were treated with either 10 μM MG132 (Selleck Chemicals) or vehicle control (DMSO) for 1 h or 3 h before harvest. For inhibition of the lysosome, TS cells were treated with either 50 μM chloroquine (CQ) (Sigma-Aldrich) or vehicle control for 16 h before harvest. Experiments for TS cell differentiation to JZ or LAB were performed as previously described (5, 6), TS cells were initially seeded in complete TS media. The following day, the cells were cultured in the absence of MEF-CM, FGF4, and heparin. Cells were differentiated for four days and were treated once daily with either vehicle control (DMSO) for JZ differentiation, or 3 μM CHIR-99021 (Selleck Chemicals) for differentiation to LAB cells.

HEK293T and COS-7 cells were a kind gift from Dr. Gary Johnson (UNC, Chapel Hill) and were cultured and transiently transfected as previously described with one modification (1, 7). HEK293T cells were cultured with 10% FetalPlex (Gemini Bio). Plasmids are detailed in Table S2.

Cell lysates, immunoprecipitation, and Western blotting

Cells were briefly disrupted and harvested in buffer supplemented with protease and phosphatase inhibitors as previously described (6, 8). Embryonic tissues were homogenized for a 5 s burst on ice in buffer with protease and phosphatase inhibitors

as described for cell lysates. Lysates were immunoprecipitated with the indicated antibodies for 1 h, after which the samples were incubated with Protein G or Protein A Sepharose for 1.5 h. Membranes from immunoprecipitations were incubated with either secondary antibody or TidyBlot Western Blot Detection Reagent (Bio-Rad). Antibodies are listed in Table S3. Western blots were developed using either Clarity Western ECL Substrate (Bio-Rad) or Clarity Max Western ECL Substrate (Bio-Rad) and were imaged using Bio-Rad Chemidoc Touch. Densitometry was performed using Bio-Rad Image Lab software.

Real-time qPCR

TS cell RNA was isolated, cDNA prepared, gene expression measured and normalized as previously described (4, 6). Primers used are indicated in Table S4.

Immunofluorescence staining, imaging, and quantitation

COS-7 cells were plated on acid washed coverslips 24 h after transfection. The following day, the cells were fixed with 3% paraformaldehyde in 1X PBS for 10 min and permeabilized with 0.1% Triton in 1X PBS for 3 min. Blocking was performed using 5% FBS in 1X PBS for 1 h at room temperature. After incubation with primary antibody, cells were washed for 30 min with 1X PBS and then incubated with DAPI (0.2 µg/ml) and secondary antibody for 1 h at room temperature. Coverslips were washed and mounted on slides with mounting media containing 90% glycerol and 10% 1 mM Tris pH 7.5. Antibodies are listed in Table S3. Images were captured using Agilent BioTek Lionheart FX microscope, 40x phase contrast objective (Agilent BioTek 1320518), and BioTek Gen5 software. Using brightfield, a minimum of 15 regions of interest (ROIs) per coverslip were selected, beacons set, and images captured. Cellular analyses were

performed using BioTek Gen5 software including total cell count per ROI. Subpopulation analyses were performed on DyLight 488-positive, Alexa 594-positive, and dual-positive subpopulations. For subpopulation analyses, minimum threshold intensities were set for DyLight 488 and Alexa 594 channels. Cells not reaching minimum thresholds were excluded. Mean DyLight 488 and Alexa 594 fluorescence intensities of subpopulations were quantified in every ROI. The mean intensities of each subpopulation in every ROI were averaged to calculate mean intensities for the entire coverslip. Images were false-colored to magenta and green using BioTek Gen5 software.

Human trophoblast differentially expressed gene analysis and data visualization

Gene expression data from bulk RNA-Seq of human TS cell-derived extravillous trophoblast (EVT) and syncytiotrophoblast (STB) cells (GSE158901) were processed using R. The EVT and STB data were isolated from the dataset, and the 'DESeq2' (1.44.0) R package was used to calculate the log2 fold change in gene expression between these cells. The Wald test was used to measure differentially expressed genes. The log2 fold change expression data was converted to fold change values prior to plotting these data using the 'ggplot2' R package.

Mouse dissections

Timed matings of WT 129/SvEv mice were performed as previously described (6). E13.5 embryos were euthanized by decapitation, head tissue quick frozen, and stored at -80°C.

Ethics statement

All experiments using animals were approved by the Institutional Animal Care and Use Committee (IACUC) at the University of Memphis. All experiments using animals were performed according to institutional and National Institutes of Health guidelines and regulations.

Statistics and analyses

Statistical analyses were performed using Prism 9 (GraphPad Software, Inc.). Densitometry of Western blots were analyzed using two-tailed Student's *t* test or ANOVA with Tukey's HSD or Dunnett's test as indicated in the figure legend. Fluorescence intensity data and qPCR data were analyzed using two-tailed unpaired Student's *t* test. Information about statistical tests used and number of replicates or individuals are defined in the figure legends. Values of * $p < 0.05$, ** $p < 0.01$, *** $p < 0.001$, and **** $p < 0.0001$ were considered statistically significant.

Table S1. Expression of Hdacs and Trafs in TS^{WT} and TS^{KI} cells.

Gene	TS^{WT}	TS^{KI}
Hdac1	49.88 ± 16.14	62.03 ± 3.78
Hdac2	8.47 ± 0.99	10.47 ± 1.08
Hdac3	12.98 ± 3.98	13.71 ± 4.38
Hdac4	2.54 ± 1.01	2.66 ± 0.59
Hdac5	12.56 ± 2.19	9.78 ± 1.40
Hdac6	28.04 ± 6.32	69.70 ± 26.95
Hdac7	5.67 ± 1.78	13.96 ± 2.93
Hdac8	0.10 ± 0.18	0.04 ± 0.06
Hdac9	0.01 ± 0.01	0.05 ± 0.03
Hdac10	0.76 ± 0.07	0.93 ± 0.32
Traf1	0.04 ± 0.03	0.11 ± 0.16
Traf2	4.44 ± 0.37	5.13 ± 0.28
Traf3	5.08 ± 0.49	5.53 ± 0.25
Traf4	26.46 ± 0.23	44.29 ± 3.28
Traf5	0.14 ± 0.01	0.35 ± 0.05
Traf6	4.91 ± 1.43	3.31 ± 0.44
Traf7	28.45 ± 3.37	16.35 ± 5.86

Transcript values are reads per kilobase million (RPKM) as measured by RNA-Seq.

Data are the mean ± range of two independent experiments.

Table S2. Plasmids and sources

Plasmid	Source/Reference
EGFP-HDAC6	Addgene, #36188<br>Gao, 2010 (9)
Flag-HA CBP	Bradney, 2003 (10)
Flag-HDAC6	Addgene, #30482<br>Kawaguchi, 2003 (11)
Flag-HDAC6 DC	Addgene, #30483<br>Kawaguchi, 2003 (11)
Flag-HDAC7	Addgene, #13824<br>Fischle, 1999 (12)
Flag-TRAF6	Nakamura, 2010 (13)
HA-TRAF4	Ye, 1999 (14)
ΔN HA-TRAF4	Ye, 1999 (14)
ΔTRAF TRAF4	Addgene, #16377<br>Sax, 2003 (15)

Table S3. Antibodies and sources

Antibody	Vendor	Part #	RRID #
Actin	Sigma-Aldrich	A4700	AB_476730
Ac-α-tubulin	Sigma-Aldrich	T6793	AB_477585
Akt1	Cell Signaling Technology	2938	AB_915788
Phospho-Akt (Ser473)	Cell Signaling Technology	9271	AB_329825
Anti-mouse IgG, HRP linked antibody	Cell Signaling Technology	7076	AB_330924
Anti-rabbit Alexa Fluor 594	Cell Signaling Technology	8889	AB_2716249
Anti-rabbit IgG, HRP linked antibody	Cell Signaling Technology	7074	AB_2099233
CBP	Cell Signaling Technology	7389	AB_2616020
Donkey anti-mouse peroxidase conjugate	Jackson ImmunoResearch Labs	715-035-151	AB_2340771
Donkey anti-rabbit peroxidase conjugate	Jackson ImmunoResearch Labs	711-035-152	AB_10015282
Flag mouse	Thermo Fisher Scientific	MA1-91878	AB_1957945
Flag rabbit	Rockland	600-401-383	AB_219374

Goat anti-mouse DyLight 488	Thermo Fisher Scientific	35503	AB_1965946
HA mouse	Gift from Dr. Gary Johnson, UNC Chapel Hill	12CA5	
HA rabbit	Santa Cruz	sc-805	AB_631618
HDAC6	Cell Signaling Technology	7612	AB_10889735
HDAC6	Bethyl Laboratories	A301-342A	AB_937899
Phospho-HSP27 (Ser82)	Cell Signaling Technology	9709	AB_11217429
Rabbit IgG polyclonal	Abcam	ab171870	AB_2687657
TidyBlot Western Blot Detection Reagent:HRP	BioRad	STAR209	
TRAF4	Cell Signaling Technology	18527	AB_2798802
Ubiquitin	Cell Signaling Technology	3933	AB_2180538
α-tubulin	Sigma-Aldrich	T9026	AB_477593

Table S4. Primer list

Gene	F Primer	R Primer
Rps11	CGCGTGGTGAATAAGGAAGC	GTAAGCACGCTCCGTCTGAA
Traf4	GCAGGAGTTTCTCAGTGAAGGA	ACTGTGGATGCAGCGGATAG

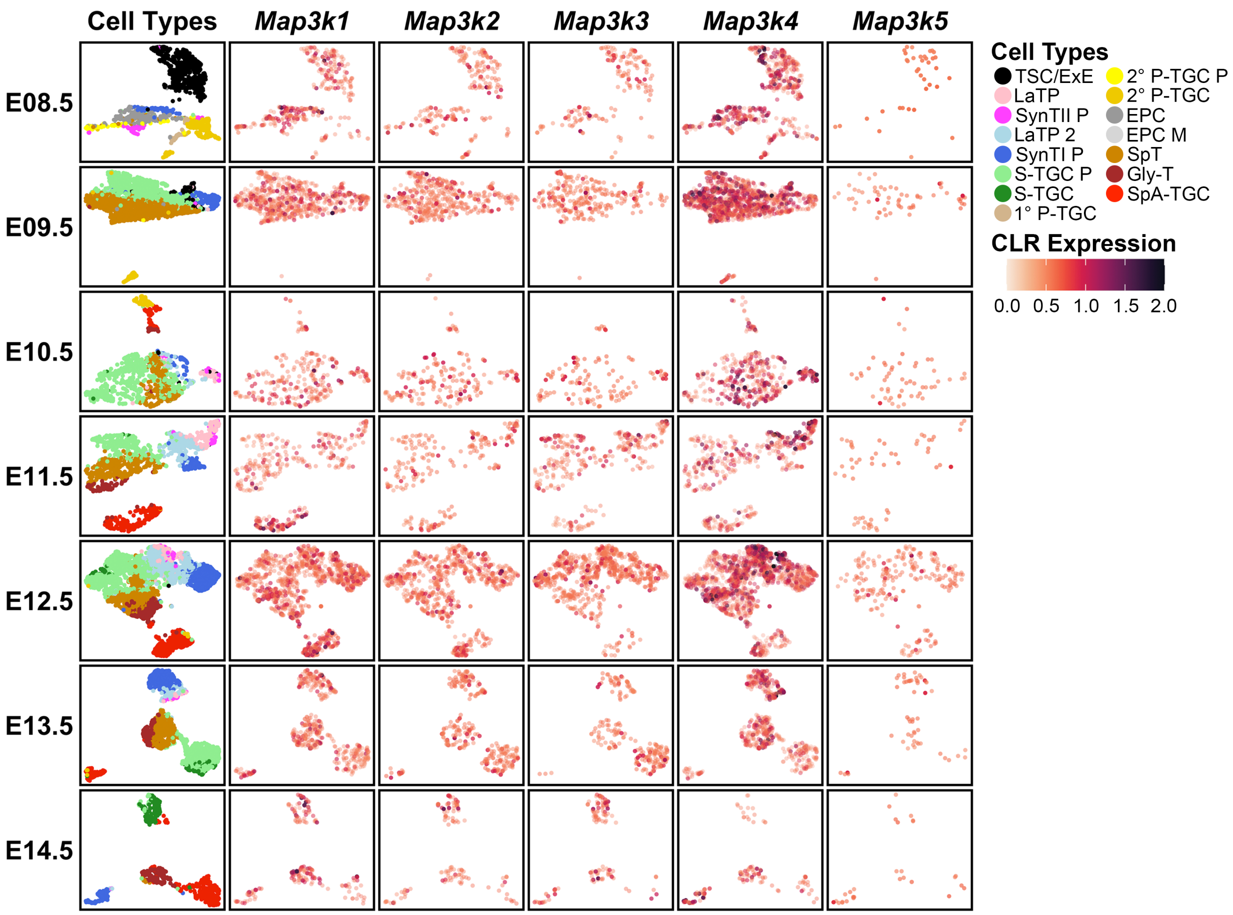

Figure S1. *Map3k4* is one of the most abundant Map3ks in the mouse placenta.

Uniform manifold approximation and projection (UMAP) plots show single-cell RNA-Seq gene expression patterns in the mouse placenta at different days of embryonic (E) development. CLR, centered log-ratio; EPC, ectoplacental cone; EPC M, EPC migratory; ExE, extraembryonic ectoderm; Gly-T, glycogen trophoblasts; LaTP, labyrinth trophoblast progenitor; P-TGC, parietal TGC; P-TGC P, P-TGC precursor; S-TGC, sinusoidal TGC; S-TGC P, S-TGC precursor; SpA-TGC, spiral-artery TGC; SpT, spongiotrophoblast; SynT, syncytiotrophoblast; SynTI P, SynT layer I precursor; SynTII P, SynT layer II precursor; TGC, trophoblast giant cell; TS, trophoblast stem; TSC, TS cell; 1°, primary; 2° secondary.

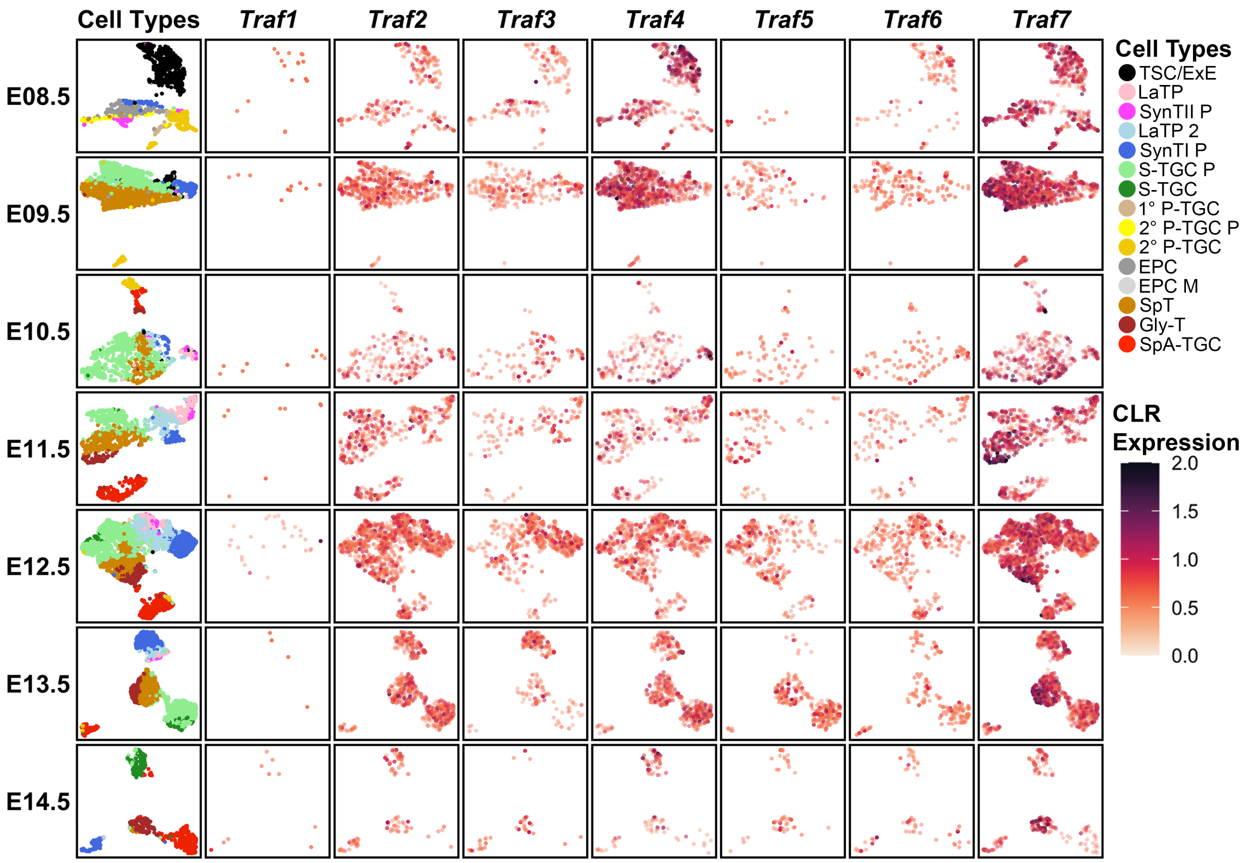

Figure S2. *Traf4* is one of the most abundant Trafs in the mouse placenta.

Uniform manifold approximation and projection (UMAP) plots show single-cell RNA-Seq gene expression patterns in the mouse placenta at different days of embryonic (E) development. CLR, centered log-ratio; EPC, ectoplacental cone; EPC M, EPC migratory; ExE, extraembryonic ectoderm; Gly-T, glycogen trophoblasts; LaTP, labyrinth trophoblast progenitor; P-TGC, parietal TGC; P-TGC P, P-TGC precursor; S-TGC, sinusoidal TGC; S-TGC P, S-TGC precursor; SpA-TGC, spiral-artery TGC; SpT, spongiotrophoblast; SynT, syncytiotrophoblast; SynTI P, SynT layer I precursor; SynTII P, SynT layer II precursor; TGC, trophoblast giant cell; TS, trophoblast stem; TSC, TS cell; 1°, primary; 2° secondary.

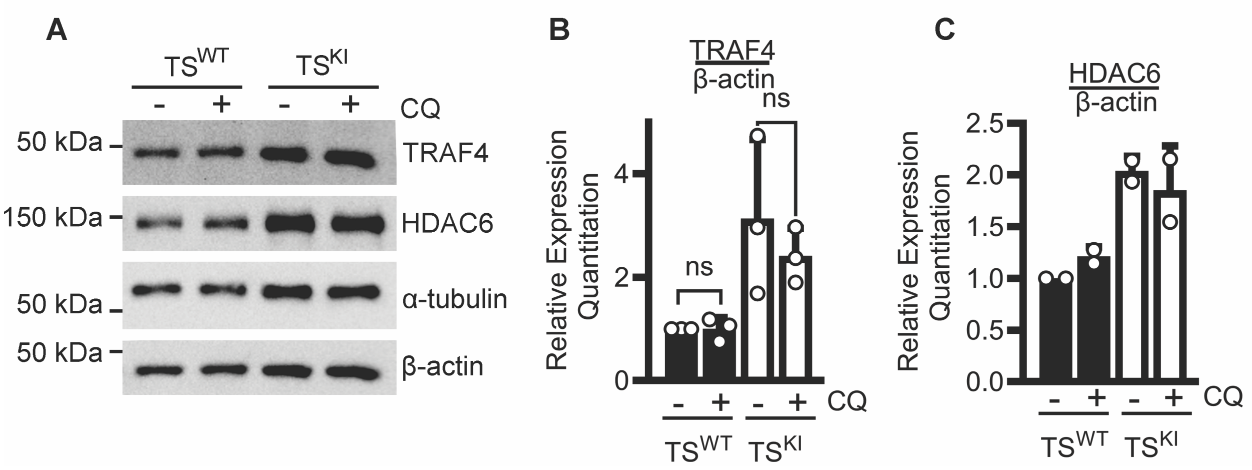

Figure S3. Inhibition of the lysosome does not impact TRAF4 or HDAC6 expression.

A-*C*, TS^{WT} and TS^{KI} cells were treated with either vehicle control or 50 μM chloroquine (CQ) for 16 h prior to harvest. *A,* representative Western blots are shown. *B*, densitometry analyses of TRAF4 expression show the mean ± SD of three biologically independent experiments. *C*, densitometry analyses of HDAC6 expression show the mean ± range of two biologically independent experiments. Student's *t* test; ns, not significant. KI, kinase-inactive; TS, trophoblast stem.

Supplemental references

1. Mobley, R. J., Raghu, D., Duke, L. D., Abell-Hart, K., Zawistowski, J. S., Lutz, K. *et al.* (2017) MAP3K4 Controls the Chromatin Modifier HDAC6 during Trophoblast Stem Cell Epithelial-to-Mesenchymal Transition. *Cell Rep.* **18**, 2387-2400
2. Shendy, N. A. M., Raghu, D., Roy, S., Perry, C. H., Safi, A., Branco, M. R. *et al.* (2020) Coordinated regulation of Rel expression by MAP3K4, CBP, and HDAC6 controls phenotypic switching. *Commun. Biol.* **3**, 475
3. Abell, A. N., Granger, D. A., Johnson, N. L., Vincent-Jordan, N., Dibble, C. F., and Johnson, G. L. (2009) Trophoblast stem cell maintenance by fibroblast growth factor 4 requires MEKK4 activation of Jun N-terminal kinase. *Mol. Cell Biol.* **29**, 2748-2761
4. Raghu, D., Mobley, R. J., Shendy, N. A. M., Perry, C. H., and Abell, A. N. (2019) GALNT3 Maintains the Epithelial State in Trophoblast Stem Cells. *Cell Rep.* **26**, 3684-3697 e3687
5. Abell, A. N., Jordan, N. V., Huang, W., Prat, A., Midland, A. A., Johnson, N. L. *et al.* (2011) MAP3K4/CBP-regulated H2B acetylation controls epithelial-mesenchymal transition in trophoblast stem cells. *Cell Stem Cell* **8**, 525-537
6. Perry, C. H., Mullins, N. A., Sweileh, R. B. A., Shendy, N. A. M., Roberto, P. A., Broadhurst, A. L. *et al.* (2022) MAP3K4 promotes fetal and placental growth by controlling the receptor tyrosine kinases IGF1R/IR and Akt signaling pathway. *J. Biol. Chem.* **298**, 102310
7. Chen, C., and Okayama, H. (1987) High-efficiency transformation of mammalian cells by plasmid DNA. *Mol. Cell Biol.* **7**, 2745-2752
8. Shendy, N. A. M., Broadhurst, A. L., Shoemaker, K., Read, R., and Abell, A. N. (2021) MAP3K4 kinase activity dependent control of mouse gonadal sex determination. *Biol. Reprod.* **105**, 491-502
9. Gao, Y. S., Hubbert, C. C., and Yao, T. P. (2010) The microtubule-associated histone deacetylase 6 (HDAC6) regulates epidermal growth factor receptor (EGFR) endocytic trafficking and degradation. *J. Biol. Chem.* **285**, 11219-11226
10. Bradney, C., Hjelmeland, M., Komatsu, Y., Yoshida, M., Yao, T. P., and Zhuang, Y. (2003) Regulation of E2A activities by histone acetyltransferases in B lymphocyte development. *J. Biol. Chem.* **278**, 2370-2376
11. Kawaguchi, Y., Kovacs, J. J., McLaurin, A., Vance, J. M., Ito, A., and Yao, T. P. (2003) The deacetylase HDAC6 regulates aggresome formation and cell viability in response to misfolded protein stress. *Cell* **115**, 727-738
12. Fischle, W., Emiliani, S., Hendzel, M. J., Nagase, T., Nomura, N., Voelter, W., and Verdin, E. (1999) A new family of human histone deacetylases related to Saccharomyces cerevisiae HDA1p. *J. Biol. Chem.* **274**, 11713-11720
13. Nakamura, K., Kimple, A. J., Siderovski, D. P., and Johnson, G. L. (2010) PB1 domain interaction of p62/sequestosome 1 and MEKK3 regulates NF-kappaB activation. *J. Biol. Chem.* **285**, 2077-2089
14. Ye, X., Mehlen, P., Rabizadeh, S., VanArsdale, T., Zhang, H., Shin, H. *et al.* (1999) TRAF family proteins interact with the common neurotrophin receptor and modulate apoptosis induction. *J. Biol. Chem.* **274**, 30202-30208

15. Sax, J. K., and El-Deiry, W. S. (2003) Identification and characterization of the cytoplasmic protein TRAF4 as a p53-regulated proapoptotic gene. *J. Biol. Chem.* **278**, 36435-36444
